# Admixed gene expression models expand molecular and neurological insights into 6 major psychiatric disorders

**DOI:** 10.21203/rs.3.rs-6229829/v1

**Published:** 2025-03-21

**Authors:** Xavier Bledsoe, Nathan Watkins, Tavian Bowen-Moore, Eric R. Gamazon

**Affiliations:** Medical Scientist Training Program, Vanderbilt University, Nashville, TN; Vanderbilt Genetics Institute, Vanderbilt University Medical Center, Nashville, TN; Chapman University, Orange, CA; Gonzaga University, Spokane, WA; Vanderbilt Genetics Institute, Vanderbilt University Medical Center, Nashville, TN; Vanderbilt Memory & Alzheimer’s Center, Nashville, TN

## Abstract

Our understanding of the influence of ancestral background on genetically determined expression remains limited, especially when gene expression models are applied to studies from different or multiple populations. We performed transcriptome wide association studies (TWAS) in 6 different psychiatric conditions, leveraging gene expression models trained in cohorts with different proportions of African, European, and Indigenous American genetic ancestries. For comparison we repeated each TWAS using a model trained in individuals of predominantly European ancestry. We identified 1,416 statistically significant TWAS associations (FDR p < 0.05) across the 6 diagnoses, of which 62% were uniquely detected by the admixed gene models. We observed > 92% correlation in the gene-level effects on disease risk, a statistic that remained robust for TWAS results that only reached statistical significance in one population. Using admixed gene expression models validated and greatly extended the yield of TWAS. The resulting transcriptomic signatures implicated neuroimaging features associated with diagnostic symptoms.

## Background

Uncovering the physiologic basis of psychiatric conditions has long been a challenge in medical science. Genome wide-association studies (GWAS) and large-scale cohort studies have heralded a major expansion in molecular associations with psychiatric conditions.^1^ Recently, transcriptome-wide association studies (TWAS) have built upon the GWAS framework by identifying imputed genetically determined gene expression measures associated with diagnoses.^2–4^ Improvements in TWAS implementation offer further enhancements for evaluation of psychiatric disease mechanisms.

One such improvement is the expansion of ancestral diversity in the training data for TWAS models. The TWAS methodology relies on pre-existing reference panels which detail the relationship between single nucleotide polymorphisms and RNA transcript quantity.^5^ Model training transforms these data into predictive models^4^ which can then be used by formal methods to quantify associations between genetically regulated gene expression (GReX) and disease.^6,7^

Traditionally, TWAS models have been trained primarily on individuals of European ancestry.^5,8,9^ It has been shown that the predictive performance of models trained in individuals of European ancestry is diminished when these models are applied to individuals from different populations.^8,10^ As new GWAS meta-analyses for psychiatric conditions from ancestrally diverse study participants are published, the development and implementation of admixed TWAS models may improve interpretation of GWAS findings.

Recently, Kachuri et al generated TWAS models from two consortia enriched for individuals of admixed ancestry: the Genes-environments and Admixture in Latino Asthmatics (GALA II) and Study of African Americans, Asthma, Genes, and Environments (SAGE).^11^ These models were trained on data from 2,733 individuals who self-identify as African American, Mexican, Puerto Rican, or other Latino American. The individuals studied in each model demonstrate different proportions of genetic admixture across African, American, and European superpopulations^11^. We hypothesize that this increased genetic diversity will enable identification of novel gene-level associations in the context of psychiatric disease. However, it is not yet clear how discordance in ancestry between GWAS cohorts and TWAS models affect the disease-GReX associations identified by TWAS.

Here we used the G2S models to perform TWAS for psychiatric conditions. To avoid trait-specific findings, we performed the TWAS in parallel on 6 major psychiatric conditions: major depressive disorder, alcohol use disorder, attention deficit hyperactivity disorder, bipolar disorder, post-traumatic stress disorder, and schizophrenia.^12–18^ We then performed TWAS for each condition using a whole blood gene expression model trained in individuals of predominantly European ancestry from the Genotype-Tissue Expression (GTEx) consortium.^5^

Prior to interpretation of the resulting gene-level associations, we first performed an in-depth statistical assessment into the comparative performance of the G2S and GTEx models when applied to GWAS of varying ancestries. We found substantial heterogeneity in the SNP features for the same genes across different models. This heterogeneity was reflected in poorly correlated p-values for the gene level associations present in both G2S and GTEx derived TWAS findings. When we examined the effect size estimates themselves, however, we observed a high degree of correlation. The estimated effect of GReX on disease risk was largely stable against variation in model ancestry, disease type, GWAS ancestry, SNP features, and SNP weights, indicating that SNP level differences between populations converge on similar gene-level targets in the context of psychiatric disease. Lastly, we show the value of the admixed TWAS approach by presenting each newly expanded set of gene level associations and imputing the neurological consequences of disease-associated transcriptomic signatures. We provide evidence that the application of ancestrally diverse gene expression models to psychiatric GWAS’ replicates and substantially expands the set gene-level and brain-level associations previously obtained from European-ancestry models.

## Methods

### GWAS summary statistics

We utilized GWAS meta-analyses summary statistics from the Psychiatric Genetics Consortium (PGC) for all 6 psychiatric diseases.^12–18^ For each condition, we selected the most recent large multi-ancestry GWAS, with the exception of BD1, ADHD, and AUD for which the largest GWAS included individuals exclusively of European descent (Table S1). For select GWAS analyses, summary statistics for both the full meta-analysis and subsets of ancestry-specific data were available. In such instances, we analyzed all available datasets separately.

### Gene expression models

We leveraged 4 PrediXcan (whole blood gene expression) models trained on data from the GALAII/SAGE (G2S) cohort studies. Three models are specific to self-reported ancestry: African American, Mexican American, and Puerto Rican. G2S includes individuals of ‘Other Latino’ ancestry; however, with only 299 individuals, this group was too small to create an independent training model. Their genetic and transcriptomic data are included in the fourth model, referred to as ‘All whole blood’. This model includes all data across the full cohort in a single gene expression prediction model. These models are publicly available on Zenodo at https://zenodo.org/doi/10.5281/zenodo.6622367.

For the predominantly European whole blood gene expression model we accessed the GTEx data from the public Zenodo repository https://zenodo.org/doi/10.5281/zenodo.3842263. The model is trained on GTEx v8 data. GTEx version 8 contains 15,201 RNA-sequencing samples quantified from 49 tissues of 838 postmortem donors. 85.3% of the donor population (715) was identified as European American, with additional demographics including 12.3% African American (103), 1.4% Asian American (12), and 1.9% reporting Hispanic or Latino ethnicity (16). 66.4% of donors (557) were male and 33.5% were female (285).^5^ We selected the PrediXcan model for whole blood run to best match the tissue used in the creation of the GALAII/SAGE models which was also whole blood.

### PrediXcan TWAS analyses

We performed TWAS on all GWAS summary statistics for the 6 selected traits obtained from the PGC. We used S-PrediXcan command line tool from the MetaXcan package using the 5 different gene expression models described above for each set of summary statistics.^19^ Each pairing of GWAS summary statistics and TWAS gene expression model is assessed individually. Prior evidence points to the presence of large inversion structural variants in the chromosome 17 and 8 regions that are disproportionately detected in European genetic ancestry and also violate statistical assumptions regarding LD underlying the TWAS approach.^20^ Consistent with prior practice, we removed the results from these regions that are predicted by the European dominant model.^21^ In all models, we removed findings from the MHC region due to similar issues in structural complexity.^22^

### TWAS multiple testing correction

We performed multiple testing correction using the Benjamini-Hochberg false discovery rate (FDR < 0.05). We assessed TWAS results for each disease and gene expression model pairing as independent studies.

### Mean correlation for each disease/ancestry pair as a function of p-value threshold

We performed TWAS of the ancestry specific GWAS datasets using the 5 gene expression models described above. At each TWAS p-value threshold, we calculated the Pearson correlation of effect size estimates with the corresponding discovery TWAS. We then identified the mean correlation for each disease/ancestry pair at each p-value threshold.

### Cross-model SNP feature comparison

We compared the SNP features in the G2S models against the GTEx whole blood model. We first subset both G2S models and GTEx models with the SNPs from the GWAS study. We then annotated each gene as ‘G2S’ if there are only SNP features for that gene in a G2S model. We made a similar annotation for GTEx. Some genes had SNP features in both models. We annotated these genes as either ‘shared_distinct’ when the SNP features did not overlap between the two studies or ‘shared_overlapping’ if there was at least one SNP shared between the two. We defined an additional gene-level annotation depending on whether the gene-disease association reached statistical significance in none of the models, G2S only, GTEx only, or both. Stratifying genes across the model statistical significance and SNP sharing, we calculated the correlation of the normalized effect size estimates (zscore) as calculated in the G2S model and the GTEx model.

### Gene annotation by model specificity

The full set of genes evaluated by GTEx is not identical to the set of gene evaluated by G2S. Certain genes are characterized only in one model but not the other. We annotated each gene according to its presence across models. Genes that were tested in any of the 4 G2S models but not GTEx were classified as ‘G2S only’ while those tested in GTEx whole blood but none of the G2S genes were classified as ‘GTEx only’. The genes that were tested in both GTEx and at least one G2S gene were classified as ‘co-tested’.

### Transcriptomic similarity across psychiatric diseases

For each disease/ancestry pair, we curated a list of effect size estimates of all associated genes that passed the significance threshold (FDR < 0.05). We then calculated the Pearson correlation of effect size estimates for shared genes across diseases for each gene model. These data were visualized by correlogram.

### NeuroimaGene mapping of disease genes to brain structure

We used the NeuroimaGene package in R to identify neuroimaging features implicated by the transcriptomic signatures of each disease.^23^ The NeuroimaGene R package enables the user to test for statistically significant associations between GReX measures and MRI-based neuroimaging derived phenotypes (NIDPs). These NIDPs were characterized in European individuals from the UK biobank and encode measurements of cortical area, volume, and thickness as well as subcortical volumes. The repository of GReX-NIDP associations was generated via application of JTI-PrediXcan to GWAS of over 3,500 NIDPs in the UK Biobank. Full description of the data and methods can be found in the original publication.^21^ We used the NeuroimaGene method to extend the interpretability of our disease TWAS by relating molecular correlates of disease to brain features which may carry psychiatric or psychological import. We first identified transcriptomic correlates with each of the 6 diseases. To assess the broadest single set of results from the G2S models, we subset the TWAS findings to those derived from the aggregate whole blood model (All whole blood). We then used NeuroimaGene to identify the neuroimaging features in the Desikan cortical atlas and ‘Subcortex’ subcortical atlas that are associated (Benjamini-Hochberg FDR < 0.05) with expression of each gene set^21,23^. We then compared the number of NIDPs from the application of the G2S and GTEx models and quantified the relative increase from the use of admixed models.

## Results

### A majority of TWAS findings derive from admixed models

**Figure 1:**
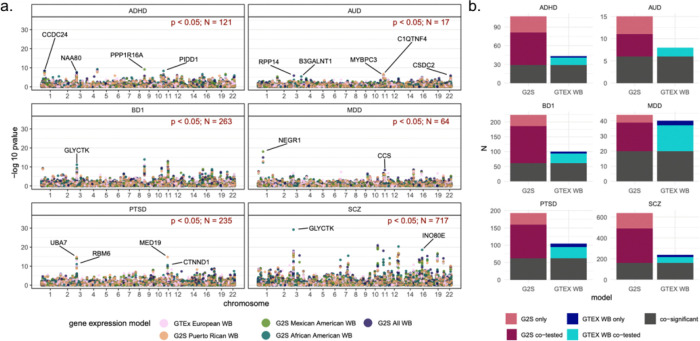
TWAS of 6 major psychiatric conditions. A. Manhattan-style plot of gene level associations with each psychiatric condition displayed. Points are colored according to the gene expression model in which the gene-disease association was identified. The top associations for each disease plot are annotated by gene name. The number of genes surpassing a false discovery rate corrected p-value of 0.05 is annotated in red in the upper right quadrant of each plot. B. Distribution of FDR-significant gene level associations for each disease according to the gene expression model. All 4 G2S models are aggregated into the G2S column. Genes that were significant in both GTEx and G2S are colored dark grey. Those that were significant in G2S only but tested in both models are in fuchsia. The co-tested genes that are significant only in GTEx are in aqua while those genes that were both uniquely tested by and significant in G2S and GTEx are colored pink and blue respectively. ADHD-Attention deficit hyperactivity disorder; AUD-Alcohol use disorder; BD1-Bipolar disorder 1; MDD-Major depressive disorder; PTSD-Post traumatic stress disorder; SCZ-Schizophrenia; G2S-GALAII/SAGE; GTEX-Genotype tissue expression consortium; WB-Whole Blood.

We identified 1,416 statistically significant associations (FDR p < 0.05) between GReX and all 6 psychiatric diagnoses (Fig. 1a, S1). Most of these associations derived from analyses of bipolar disorder 1 (263), and schizophrenia (717), with the rest from ADHD (121), major depressive disorder (64), alcohol use disorder (17), and PTSD (235). A majority of associations (62%) were uniquely detected by models trained on individuals of African American, Latino, Puerto Rican, and Mexican self-reported ancestry. This stands in contrast to the 14% of gene-level associations specific to the majority European model trained in GTEx. The high performance of the G2S models relative to GTEx remains true for bipolar disorder 1, alcohol use disorder, and ADHD, which were all derived from GWAS in individuals of exclusively European genetic ancestry^12,13,17^.

### Disease TWAS associations differ based on ancestry background of gene models

We observed that only 24% of all gene-level associations passed TWAS significance thresholds using both predominantly European and admixed models. On assessment, 79% (939) of significant TWAS genes were tested in both GTEx and G2S (Fig. 1b). Of the other 247 significant genes, 213 were only assayed by G2S while 34 were only assayed by GTEx. The 79% of co-tested genes contrasts with the 24% of significant gene-level associations that replicated across models. Because the same GWAS summary statistics are used for the different TWAS, the remaining differences in gene-level associations must arise from incongruities in the SNP features for the G2S and GTEx models.

### Gene-level TWAS associations demonstrate high correlation of effect sizes

**Figure 2:**
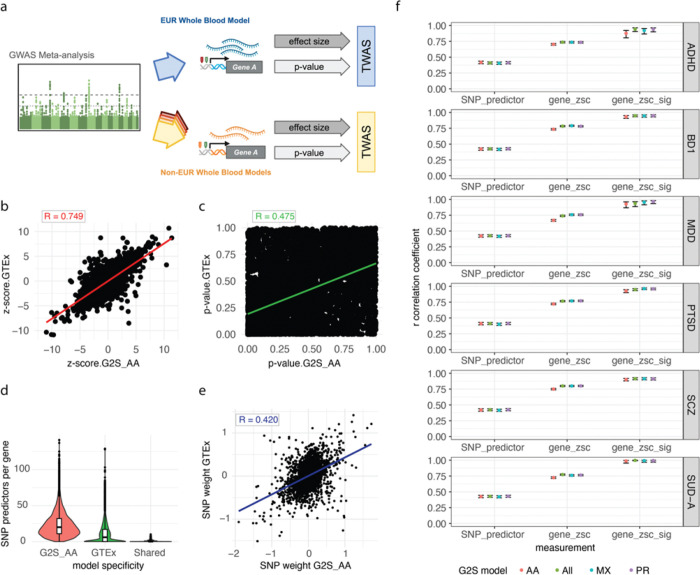
Quantitative analysis of SNP feature and gene-level data. a. Diagrammatic overview of TWAS outputs. b. Comparison of estimated effect sizes of GReX on SCZ as called by the African American (AA) G2S and GTEx models. The x-axis shows the normalized effect size of GReX on SCZ risk in G2S. The y-axis position of a point represents the normalized effect size of that same gene-disease association as identified in the GTEx model. The trendline represents a linear regression with the correlation of points across the two models recorded in the upper left quadrant. These associations are not filtered for significance. c. Comparison of p-values for GReX effects on SCZ as called by the AA G2S and GTEx models. The x-axis shows the p-value for the GReX effect size estimate on SCZ risk in G2S. The y-axis position of a point represents the p-value for the effect size estimate of that same gene-disease association as identified in the GTEx model. The trendline represents a linear regression with the correlation of points across the two models recorded in the upper left quadrant. These associations are not filtered for significance. d. Distribution of the number of SNP features per gene unique to the G2S AA training model in red, GTEx training model in green, and those that are shared in blue. Boxplots represent median values and the interquartile range. e. Comparison of SNP feature effect sizes (zscore) matched by gene across the AA G2S and GTEx models. The x-axis shows the weight of a SNP feature on gene expression in G2S. The y-axis position of a point represents the weight of that same SNP-gene association as identified in the GTEx model. The trendline represents a linear regression with the correlation value recorded in the upper left quadrant. f. Study-wide correlation of G2S vs GTEx statistics for SNP prediction weight, global GReX effect size, and significant GReX-disease association effect sizes. Points are colored according to the G2S model that is being compared against GTEx. The y-axis represents the correlation coefficient. Error bars represent the 95% confidence interval. Panels are divided according to the disease under analysis. AA-African American; MX-Mexican American; PR-Puerto Rican; ZSC-zscore

Accordingly, we examined the TWAS effect size estimates across G2S and GTEx. Each gene-level association is characterized by an estimated effect size of GReX on disease risk (Fig. 2a). For all genes and models, the correlations in GReX-disease effect sizes ranged from 0.66 to 0.80 with a mean correlation of 0.76 (Fig. 2b, Figure S2). This high correlation in effect sizes rose to an average of 0.93 when we considered only those genes that were called significant in at least one of the models (Figure S3). For the genes significant in both models, the effect sizes obtained a correlation of 0.99 (Table S2). Notably, for the genes that were called significant in either a G2S model or GTEx but not both, the effect sizes still obtained a mean correlation of 0.88 (Table S2). The 24% replication in significant findings was thus not explained by differences in effect sizes between G2S and GReX. The effect sizes matched with a high degree of correlation.

### Gene-level TWAS associations demonstrate high p-value heterogeneity

We calculated the correlation in p-values across G2S and GTEx associations. Across all genes, we obtained a mean correlation coefficient of 0.50 (Fig. 2c, S4). Restricting the set of analyzed genes to those significant in at least one TWAS resulted in a mean correlation coefficient of −0.04 (0.32 for shared significant genes; Figure S5). More significant associations demonstrated lower correlation in p-values. This low correlation in significance statistics is much more consistent with the low replication of significant gene-level associations across G2S and GTEx.

We highlight an association between *APPL2* and bipolar disease 1 as a representative example. The normalized effect size estimates for this association in GTEx whole blood and the aggregate G2S whole blood model are similar in magnitude and direction (−2.05 and − 3.22 respectively). While the effect size estimates are similar, the association reaches statistical significance in the aggregate G2S model only (P_FDR_ = 0.044) but not in GTEx (P_FDR_ = 0.29). *APPL2* has been implicated in exome sequencing of individuals with bipolar disorder 1^24^ and functions as a molecular regulator for the mania-associated *DISC1* locus^25^. Detected in G2S but not in GTEx, these data provide the first evidence associating GReX of *APPL2* with risk of bipolar disorder.

### G2S and GTEx models rely on largely distinct sets of SNP features

We next assessed if the differences in SNP features across G2S and GTEx informed the high p-value discordance of the gene-level associations. We identified the intersecting set of SNPs that were both reported in the GWAS and used in each gene expression model. The G2S models used a median of 28 SNPs per gene while GTEx used a median of 8 (Fig. 2d, S6). The average number of GWAS-interrogated SNPs shared across both models per gene was 0.90. In 56% of genes tested, no SNPs were shared between the G2S and GTEx models. The G2S models thus used more SNPs in gene expression prediction and used largely different sets of SNPs. Both the G2S and GTEx variants were obtained using whole genome sequencing.

### Cross-ancestry heterogeneity exists in SNP features of gene expression

Higher weight magnitude for a SNP feature implies that the SNP predicts a greater change in gene expression. Across diseases, only 1.8% of SNP features were shared for the same genes across the models (Fig. 2d, S6). We assessed the correlation in weights assigned to these SNP features across G2S and GTEx models. Correlations in the SNP weights ranged from 0.40–0.43 across diseases and models (Fig. 2e, S7). While G2S and GTEx used largely different sets of SNP features, even when they use the same SNP feature, the assigned weights were only modestly correlated. We thus observed high correlation in effect size estimates for all gene level associations in the presence of low sharing and low similarity of SNP weights (Fig. 2f).

### Broad differences exist across prediction models

**Figure 3:**
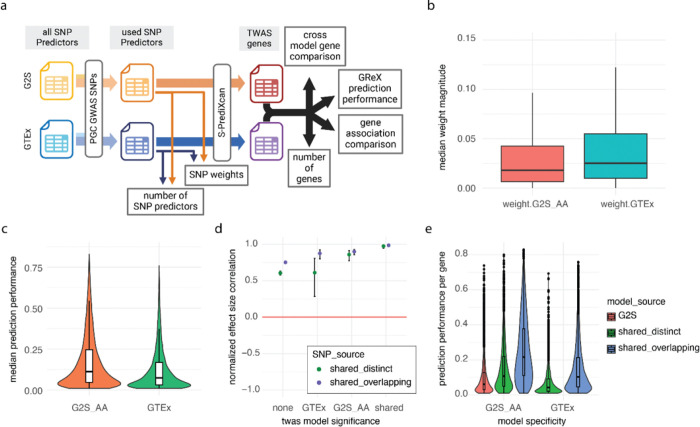
Relationships between SNP features and gene expression a. Visual overview of GWAS, SNP, and gene-based analyses. b. Comparison of effect size estimate magnitudes of SNP features on gene expression in SCZ analysis between the African American G2S model and GTEx. Each box plot shows the median and the interquartile range. c. Comparison of prediction performance of SNP features on gene expression in SCZ analysis between the African American G2S model and GTEx. Each box plot shows the median and the interquartile range. d. We plot the correlation in effect size estimates for gene level associations according to which models called the gene-level association as significant (FDR < 0.05). We then divided the genes into those that included overlapping SNP features (shared_overlapping) and genes that had completely distinct sets of SNP features across GTEx and G2S (shared_distinct). Data points represent the Pearson correlation and error bars represent the 95% confidence interval. e. Distribution of the prediction performance statistics (*r*^2^) per gene according to the SNP feature source for the G2S AA model and the GTEx model. The prediction performance statistics are presented both for the G2S AA model and the G2S GTEx model along the x-axis. Genes with SNP features only in the G2S system are presented in red. Genes with non-overlapping SNP features in both G2S AA and GTEx models are shown in green. The genes with at least one shared SNP predictor across both G2S AA and GTEx models are shown in blue.

Beyond the correlation of weights, the performance of models could be influenced by the distribution of SNP features, the accuracy of prediction performance, and other variables (Fig. 3a). As was previously noted, the G2S models used a median of 28 SNP features per gene compared to the median of 8 in GTEx. We also observed that the median weight of each SNP feature on its gene was less in G2S than GTEx (Fig. 3b). The result of differing SNP sets on gene expression imputation was re ected in the prediction performance statistic of each model. G2S obtained a median *r*^2^ of 0.157 while GTEx was significantly lower at 0.124 (Fig. 3c, S8). Conversely, for genes captured by both G2S and GTEx, the *r*^2^ was greater in G2S models (Figure S9). Collectively the G2S models predicted gene expression using a greater number of SNPs and more low-weight SNPs than the GTEx model. Regarding the SNP features shared between G2S and GTEx, the weights assigned by G2S were generally higher than those in GTEx (Fig. 2e, S10). Thus, there are systemic differences between the SNP features identified in GTEx vs those derived from populations of African American, Mexican American, and Puerto-Rican individuals.

### Cross-model TWAS correlations remain high despite distinct SNP features

The high effect-size correlation is not intuitive given the systemic differences in the SNP predictors between models. Here we assessed the null hypothesis that the convergent effect size estimates are due to shared, high impact SNP predictors used in both G2S and GTEx. To test the null, we first curated all genes with models in both G2S and GTEx. We then divided these genes into two categories depending on if the sets of SNP features for the gene are distinct (shared_distinct) or if there was at least one overlapping SNP between the predictor sets (shared_overlapping). We next stratified genes according to their TWAS significance in the G2S and GTEx based analyses. Lastly, we calculated the correlation coefficient for TWAS effect size estimates and p-values. The gene level associations derived from non-overlapping sets of SNP features demonstrated correlation coefficients that were lower but similar in range to the gene level associations derived from overlapping sets of SNP features. While sharing SNP features did increase the correlation of TWAS effect size estimates, correlations greater than 0.84 and 0.9 were still observed for genes that are significant in one or both models respectively but had no intersecting SNPs (Fig. 3d, S11). Notably these findings did not replicate for correlations of p-values, where high discordance persisted across diseases and subcategories (Figure S12).

### SNP sharing predicts gene expression with greater prediction accuracy

The *r*^2^ cross validation measure represents the correlation between predicted gene expression and measured gene expression from the training sets in GTEx and G2S.

Regardless of whether the *r*^2^ cross validation is assessed in GTEx or G2S, the genes with the greatest predictive accuracy were those classified in both GTEx and G2S models with overlapping SNP predictors (Fig. 3e, S13). Additionally, even when the SNP predictor sets were fully distinct, these dual-classified genes still demonstrated greater *r2* cross validation than genes called significant by only a single model.

### Parallel ancestry-concordant TWAS recapitulate high correlations in effect size estimates

**Figure 4:**
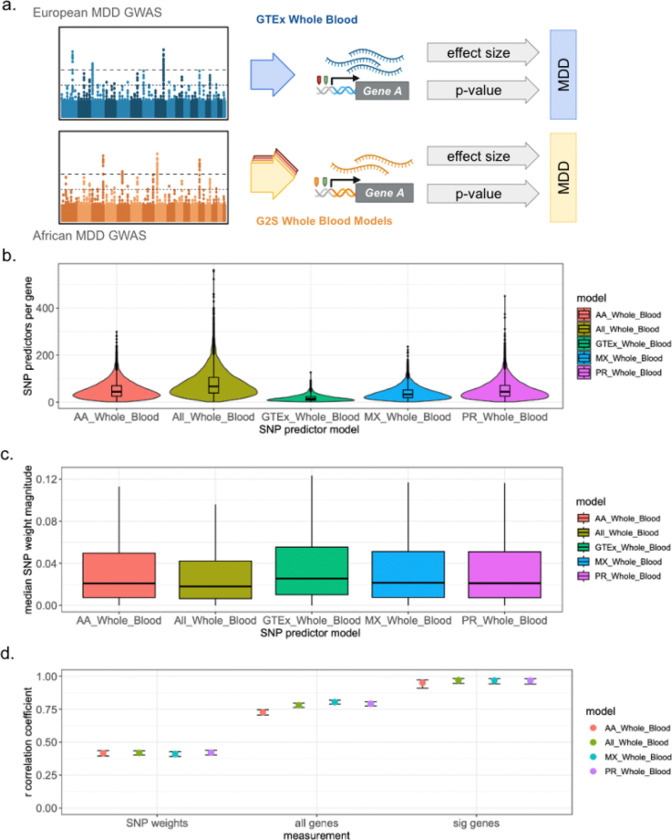
Cross-ancestry analysis of MDD TWAS a. Schematic of parallel TWAS analyses. We perform a TWAS of MDD using GTEx models applied to GWAS summary statistics from a uniquely European cohort. In parallel, we perform TWAS of MDD using the G2S models applied to GWAS summary statistics from a predominantly African ancestry cohort. b. The number of SNP features per gene used by each model in the TWAS of MDD from the pertinent GWAS analyses. c. The y-axis represents the median weight of SNP features on gene expression for all genes across the different models. Each boxplot represents the interquartile range. d. Correlation of G2S vs GTEx statistics for SNP prediction weight, global GReX effect size estimates, and significant GReX-disease association effect size estimates. Points are colored according to the G2S model that is being compared against GTEx. The y-axis represents the correlation coefficient. Error bars represent the 95% confidence interval.

Three multi-ancestry GWAS meta-analyses (PTSD, SCZ, and MDD) provided summary statistics stratified by ancestry. We performed TWAS on each subset of GWAS summary statistics using the G2S and GTEx models (Figure S14). Analyses of the African and Indigenous American subsets were not suficiently powered to identify any significant gene-level associations for any of the conditions (Figure S15). To perform parallel, ancestry concordant TWAS analyses, we used two different meta-analyzed GWAS of MDD performed by the PGC. The first is described above and included individuals of African (36%), East Asian (26%), East Asian (6%) and Hispanic/Latin American (32%).^14^ The second is a GWAS meta-analysis in individuals of only European descent (Fig. 4a).^26^ We performed a TWAS of the European GWAS using the GTEx whole blood model. In parallel, we performed TWAS of the multi-ancestry GWAS using the G2S models. As with the meta-analyzed data, we identified SNPs used in each of the G2S TWAS and compared them to the SNP features used in the GTEx TWAS. The G2S models consistently used more SNPs than GTEx (Fig. 4b) and included SNPs with smaller SNP weight magnitude on gene expression (Fig. 4c). Across shared SNP-gene pairs, we observed a correlation in the SNP prediction weights of approximately 0.42. Considering the estimated associations between GReX and disease, the correlation of effect size estimates with GTEx was approximately 0.75 for all shared genes detected in the G2S models. Restricting the effect size comparison to genes that were significantly associated with MDD in at least one model, we again identified correlation values of greater than 0.95 (Fig. 4d).

### Phenomic correlation analysis using transcriptomic signatures

**Figure 5:**
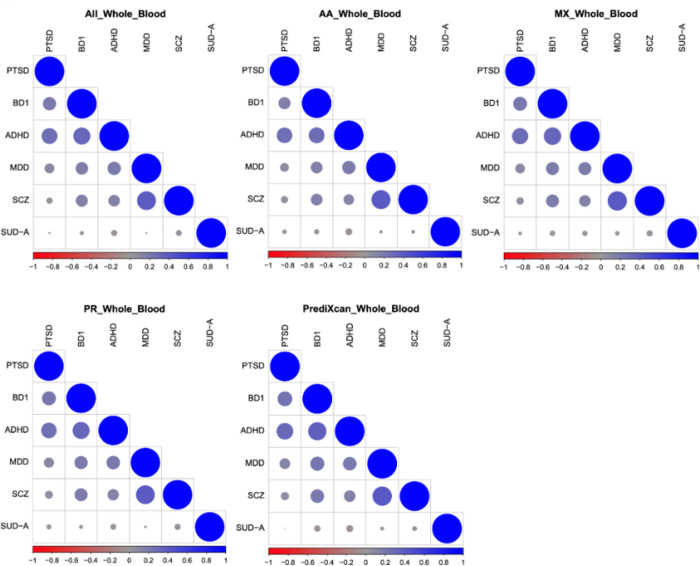
Correlograms detailing the transcriptomic correlations across 6 major psychiatric disorders according to the G2S and GTEx whole blood models. The axes are categorical representations of each psychiatric condition. The size and color of dots at the intersection of traits describe the transcriptomic correlation of effect size estimates across all tested genes for both traits.

In the analyses up to this point, we treated each disease as its own entity and compared the details of the different models within the disease. Demonstrating similarities in performance within diseases does not inform how the models will perform across diseases. The predicted relationships of transcriptomic signatures across diseases could differ depending on the model used to impute GReX. As such, we quantified the transcriptomic similarity of all 6 diagnoses. Within each ancestry model, we calculated the Pearson correlation of s across all FDR significant genes associated with each disease (Fig. 5). The rank ordering of disease pairs by correlation coefficient was similar across all diseases. The greatest transcriptomic correlation was observed between MDD and SCZ, followed by ADHD and PTSD. In all cases, we observed high correlation between BD1 and ADHD. Alcohol use disorder demonstrated little transcriptomic correlation with the other 5 diagnoses. These patterns were robust to variation in the gene expression model used across both G2S and GTEx.

### Multivariate imaging correlates of transcriptomic disease profiles

**Figure 6:**
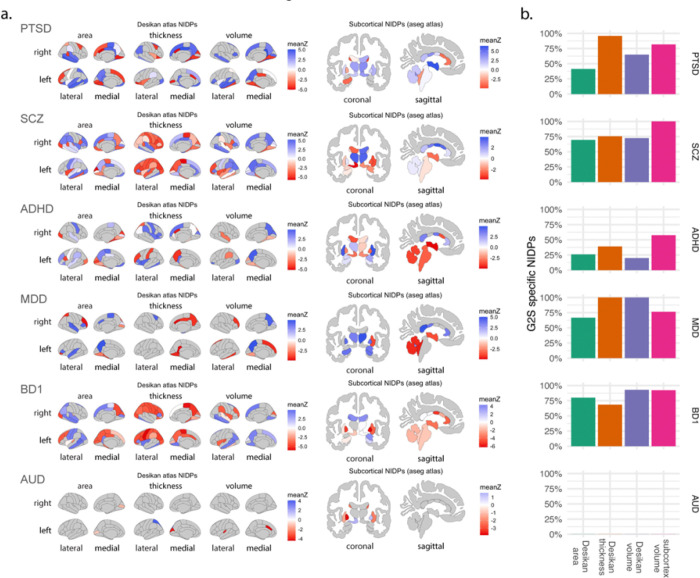
Estimated effects of TWAS gene expression on cortical and subcortical morphology. a. We use the NeuroimaGene resource to quantify the effects of GReX on the human brain according to reference data from healthy individuals. Cortical measures are reported according to the Desikan parcellation with area, thickness, and volume measures in both hemispheres shown for all 6 conditions. Subcortical findings reflect the freesurfer automated segmentation protocol and accord with volumetric measurements. The color schema indicates the mean effect size estimate of all associated disease GReX measures on the brain region. b. Certain neuroimaging findings are correlates of GReX from GTEx models while others are correlates of GReX from G2S models. We show the percentage of total detected NIDPs that are associated only with GReX from G2S models in panel b with bars split by the measurement type as shown on the x axis.

The TWAS methodology identi es statistical associations between gene expression and each psychiatric condition. This approach does not implicate aspects of organ-level biology that may mediate disease risk. Prior studies suggest that GReX changes associated with psychiatric disease have consequences on brain physiology that may affect disease risk^27^. We wanted to assess the extent to which using admixed gene expression models in TWAS improved the identification of putative biological intermediates involved in risk. We used the NeuroimaGene approach to assess the impact of the trait-associated GReX for each condition on measures of neurological structure. NeuroimaGene functions as a repository of associations between GReX and over 3,400 neuroimaging derived phenotypes. Derived from predominately healthy individuals, these associations quantify endogenous relationships between imputed gene expression and the physical structure of the brain. We used GReX measures derived from the G2S aggregate model for each disease. Using NeuroimaGene, we tested for associations between these GReX measures and NIDPs from two atlases: the Desikan cortical atlas and an automated segmentation of subcortical regions (Fig. 6).

The genes associated with BD1 and SCZ both implicated widespread cortical alterations. These findings are seen most dramatically in the negative correlation between cortical thickness and disease GReX measures. Alcohol use disorder and MDD presented with relatively little cortical targeting by trait-associated genes. This contrasted with subcortical findings where prominent effects on the bilateral putamen in alcohol use disorder and widespread subcortical involvement in MDD were observed. We noted that 59% of all NIDPs associated with trait genes were the result of genes that were significant in G2S but not GTEx. This number was highest in the volume and thickness measures associated with MDD and subcortical volumes in schizophrenia, all reaching 100%. With only 14 total NIDPs identified, alcohol use disorder presented with no G2S specific NIDPs. These statistics likely underestimate the breadth of potential information carried by the G2S genes given that the NeuroimaGene resource does not include information from diverse cohorts. It only reflects gene-level associations derived from Europeans. This limitation notwithstanding, the 489 additional NIDPs associated with the 6 conditions via G2S represent a 183% improvement over those identified by GTEx alone. As with the initial TWAS, we again observed that the use of admixed models improved association detection in context of predominately European genetic ancestries, this time in the space of neuroimaging associations.

## Discussion

We compared the results from TWAS using 5 gene expression models that differ in their genetic ancestries. To minimize capturing phenotype specific results, we repeated the analysis for 6 different psychiatric GWAS meta-analyses. Gene expression models trained on admixed populations (AFR, EUR, and AMR) generally identified more significant gene level (TWAS) associations than models trained on individuals of predominantly European ancestry. We observed this pattern when TWAS was applied to GWAS cohorts of European ancestry as well as cross ancestry meta-analyses. Our results are consistent with prior findings suggesting that the increased variance in allele frequencies can improve TWAS association power^28^.

Overall, we identified 1,416 gene-level associations with psychiatric diagnoses, of which 881 were uniquely detected in highly admixed ancestry models compared to European ancestry models. These findings suggest that there may be significant additional utility in increasing the genetic diversity of transcriptomic resources.

One previously unreported result is a significant association between ADHD and *DCHS1* in the G2S models. The association does not reach statistical significance in the GTEx whole blood analysis and has not been associated with ADHD in preexisting literature. *DCHS1* codes for a cadherin protein that is most strongly detected in the developing fetal brain^29^. Other cadherins have already been strongly associated with ADHD, supporting the plausibility of *DCHS1*.^30^ Regarding functional support, observational studies in humans as well as interventional analyses in mice indicate that perturbations of *DCHS1* leads to disruptions in cerebral cortical development.^31^ Lastly, previous data shows that gene expression models trained on non-brain tissues suffer from reduced power regarding brain-related TWAS than those trained in the brain^21^. While *DCHS1* is not associated with ADHD in the GTEx whole blood model, the association achieves statistical significance when using a gene expression model trained on the brain cortex from GTEx. This cross-tissue replication suggests that some of the inherent limitations of using whole blood models to predict psychiatric conditions may be ameliorated by using admixed gene expression models.

There was incomplete portability of statistically significant gene-level associations across G2S and GTEx models. Specifically, 76% of significant associations were either significant in a G2S model or the GTEx model but not via both. When one gene-disease association is statistically significant in one study and not another, it is not immediately clear which p-value statistic should be accorded more weight. This set of p-value discordant genes is thus of high import as they may represent false positives or novel associations with potential biological implications.

Focusing in on the associations with discrepant p-values, we observed a high degree of correlation in the effect size estimates (> 90%). This correlation is not likely to be a consequence of SNP overlap between models given that the majority of G2S/GTEx comparisons had zero overlapping SNP features per gene. When there were overlapping SNP features between genes, the weights of the features were often different, reflected by correlation coefficients near 0.4. The G2S and GTEx models (1) leveraged largely different SNPs, (2) accorded different weights to the small proportion of shared SNPs and (3) still arrived at highly correlated effect size estimates for gene-disease associations.

Our finding of convergent gene-level associations from divergent populations of SNP features speaks to an interesting question about the role of ancestry in genetic studies. Variation in the presence and frequency of different alleles across different populations is well described^32^. As a result, when SNP-based prediction algorithms such as PRS are trained in one ancestry group, they often suffer from reduced accuracy in other populations on account of these differing SNP pro les^33,34^. Similarly, GWAS of the same trait can identify different trait-associated SNP variants when performed in populations of different genetic ancestries^35,36^. The prevailing hypothesis regarding complex disease is that individuals of different populations share similar molecular disease processes which are merely being tagged by different SNPs^37,38^. Our data support this paradigm. Specifically, we observe substantial ancestry-based heterogeneity regarding which GWAS SNPs predict gene expression. The convergence of these different SNP features onto a shared set of highly correlated gene-disease associations suggests that similar transcriptomic markers of psychiatric disease are tagged by different SNPs depending on the population used for imputation reference. This is further supported by our analysis of MDD for which two different ancestry-stratified GWAS were each assessed via the TWAS methodology using ancestry concordant gene expression models.

Linkage disequilibrium is a topic that deserves special consideration in the context of this analysis. Our main findings were that (1) the highly admixed G2S models identified more associations than the predominately European GTEx model, (2) the effect size estimates of gene-disease associations were largely similar across tested ancestry groups, and (3), this sharing was robust to highly discordant SNP features across gene expression models. Linkage disequilibrium is most relevant to finding 3 and we expect that some degree of cross ancestry LD exists between SNP features from G2S and GTEx. While LD could explain a proportion of the correlation in gene-disease effect size estimates, it would not invalidate the primary result which is that the correlation exists. Should that correlation exist due to cross-ancestry LD of SNP features, such a finding would further validate the claim that there is substantial sharing of gene-disease associations across populations. While we do not assess this question here, such an analysis could be an interesting follow up to the work presented.

The full set of transcriptome associations for each disease represents a method of describing a disease entity by its molecular correlates. We compared the molecular similarities between diseases (Fig. 5). If PTSD for example possessed a different molecular profile in one ancestry relative to another, we would expect the transcriptomic relationship of PTSD to other diseases to differ. Instead, we observed a similar relationship between transcriptomic signatures of disease across all 5 models.

Lastly, we observed that the inclusion of genes identified by the admixed models increased the identified disease-relevant neuroimaging features by 183%, highlighting the potential for these associations to better inform the neurobiology of psychiatric disease.

This study has several relevant limitations. The G2S models are defined using self-reported ancestry^11^. Because admixture thresholds were not used for participant inclusion, the models cannot be treated as strictly representative of discrete genetic ancestries. European admixture increases the SNP-level similarity of the G2S and GTEx models. The same is true for GTEx given the 12% of participants who were identified as African American and the ~ 3.1% of individuals identifying as Asian American or Latino. While the result of this admixture would be to increase SNP-level similarities in gene expression models, we still observe substantial heterogeneity which then converges on shared biological intermediates including RNA transcripts and neuroimaging features. Secondly, we limit these analyses to psychiatric disease GWAS published by the Psychiatric Genomics Consortium to minimize heterogeneity in the meta-analytic methodology for the source data. Therefore, these findings are not guaranteed to extrapolate to non-psychiatric conditions. Our data include just five gene expression models covering only European, indigenous American, and African genetic ancestries. There is a tremendous genetic variation in other people groups that is not represented here. We anticipate the creation and validation of additional TWAS models to further such analyses.

## Data Availability

All data generated in the production of this manuscript can be found at Zenodo [https://zenodo.org/uploads/14889758] (temporary link to be finalized following revisions)
